# Spatiotemporal Distributions and Dynamics of Human Infections with the A H7N9 Avian Influenza Virus

**DOI:** 10.1155/2019/9248246

**Published:** 2019-02-07

**Authors:** Yongxue Chen, Yongxian Wen

**Affiliations:** College of Computer and Information Sciences, Fujian Agriculture and Forestry University, Fuzhou 350002, China

## Abstract

In 2013 in mainland China, a novel avian influenza virus H7N9 began to infect humans and had aroused severe fatality in the infected humans, followed by the annual outbreaks. By methods of GIS and kriging interpolation, we get the geographical distributions. We obtain the longitudinal characteristics of these outbreaks based on statistics and diagrams. After these spatiotemporal distributions, an eco-epidemiological model is established and analyzed. In this model, the general incidence functions, the factor of fully killed infected poultry, and the virus in environment are taken into account. Theoretical analysis shows that the endemic will be formed to a large extent once the H7N9 avian influenza virus exists in poultry. On the basis of dynamics, we explore the possible disease control measures by numerical simulations. Simulations indicate that measures of vaccination in poultry and stopping live poultry transactions are the primary choices for disease control in humans, and strengthened inhibition effects and environmental disinfections can effectively control the outbreak.

## 1. Introduction

In early 2013, the first case of human infection with the A H7N9 avian influenza virus was found in China, and subsequently the A H7N9 influenza virus was detected in the live poultry market. At that time, the A H7N9 influenza virus had no pathogenicity or low pathogenicity to poultry. But, the fatality rate of human infections with the A H7N9 virus is much higher than that of seasonal influenza infections. Since 2013, there are over 1500 reported human cases and many human deaths [1]. In early 2017, a mutant strain was found that was highly pathogenic to poultry and led to multiple poultry outbreaks [1–4]. The A H7N9 avian influenza virus has brought great harm to the development of poultry industry and the health of public in mainland China. In the spring of 2017, it seems more imperative for the epidemic prevention and control.

The outbreaks in poultry and humans have serious impact on livelihoods, economy, and the international trade. They have raised widespread concerns. The related research booms are set off in various fields, mainly in medicine, epidemiology, biology, mathematics, statistics, genetic analysis, and so on. Zhang et al. [5] and Su et al. [6] implemented genetic analyses and verified that the reduction of the N-glycosylation site at position 42 of NA was observed in some strains but most of the H7N9 viruses had no drug resistance mutations. Dong et al. [7] suggested genetic reassortment has occurred since the emergence of A H7N9 HPAI (the highly pathogenic avian influenza) viruses. Zhou et al. [8] presented the observational studies based on statistics. Zhang et al. [9] used a mathematical model, between wild and domestic birds and from birds to humans, to fit data and investigate the control measures in view of sensitivity analysis and the basic reproduction number *R*_0_ and demonstrated that closing the LPMs (the live poultry markets) was a very effective measure. Artois et al. [10] mentioned the spatial models in order to explain the relationship between the range expansion and the emergence of a highly pathogenic variant. Liu and Fang [11] constructed a SIR-SIR mathematical model, and parameters in the model were estimated. Zhang et al. [12] and Xing et al. [13] carried out the data fitting and the sensitivity analysis for the source factors and recurrence factors, respectively. Their analyses are based on two four-populations models, including migratory birds, resident birds, domestic poultry, and humans, involving the virus in environment. In some of the above research studies, the dynamical models were established but authors only gave out the numerical analysis due to the complexity of models. Guo et al. [14] proposed and analyzed an SE-SEIS avian-human influenza model based on the reported data and proved the global stability results for both the disease-free equilibrium point and the endemic equilibrium point by using a general Bendixson–Dulac theorem. Li et al. [15] obtained the basic reproduction number *R*_0_ by method of the next generation matrix, and local and global stabilities of the equilibria are proven. Mu and Yang [16] analyzed a SEIR model with latent period and nonlinear recovery rate dynamically. In [17], we established a SEM-SIR eco-epidemiological model incorporating the mutation factor, focused on the disease control measures and the production protection, and then both the global dynamic properties and the disease control suggestions are given. Observed from the reported cases and the following spatiotemporal distributions, also inspired by [12, 13], we formulate here a SEV-SIR model with a class of the virus in environment and analyze it dynamically and also concern about disease control and production protection.

In order to gain insight into the characteristics of disease transmission over the past few years, in this paper, we firstly present the temporal laws and spatial distributions of human infections in Section 2. In Section 3, we formulate a SEV-SIR human-avian eco-epidemiological model. In this model, the constant recruitment, the general incidence functions, the factor of fully killed infected poultry, and the virus in environment are considered. The dynamic analysis was presented in Section 4. In Section 5, numerical simulations are carried out to investigate the effective disease control measures and to explain the practical countermeasures. In Section 6, we end this paper with a conclusion.

## 2. The Spatiotemporal Distributions

**Figure 1:**
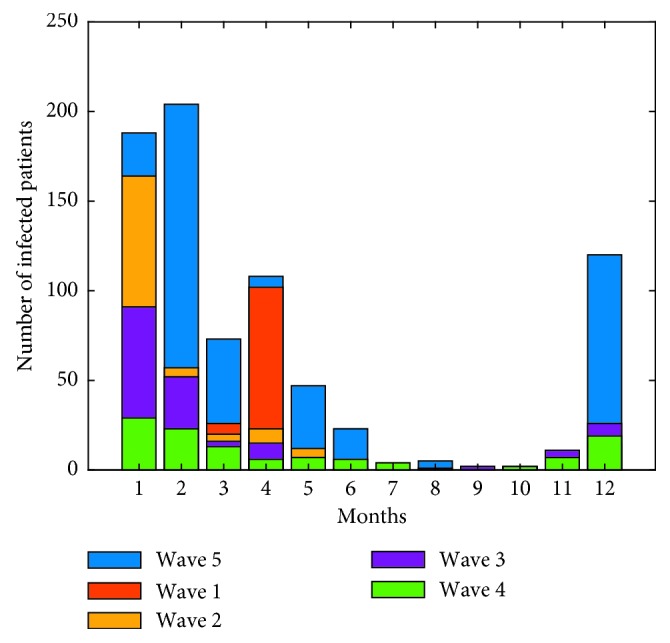
The number of infections in the past five outbreaks. The number of human infections has decreased from the second wave (yellow), the third wave (purple) to the fourth wave (green). But, infections in the fifth wave are abnormal.

The first case of human infection with the A H7N9 avian influenza virus was confirmed in February 2013. Since then, a wave of outbreak happened in the autumn and winter of each year to the spring of next year. We denote the outbreak of February 2013 to May 2013 as the first wave, the outbreak from October 2013 to May 2014 as the second wave, from October 2014 to May 2015 as the third wave, and so on. Statistics tells us that there are 133, 286, 218, 113, and 729 human cases in turn in the five past waves. In order to understand intuitively, the annual outbreaks of human infections with the A H7N9 avian influenza virus are depicted in Figure 1. We can see that the number of human infections has decreased year by year from the second wave, the third wave to the fourth wave, indicating that our control measures are effective. However, it is also clear that there are serious infections in winter in the fifth wave. The abnormal infection in the fifth wave tells us that the disease control is still very grim and needs further evaluation and exploration. In the following, we will investigate the control measures via a mathematical model.

**Figure 2:**
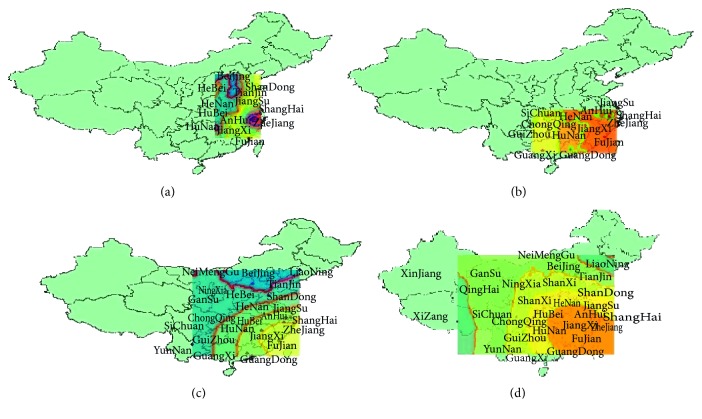
The spatial distributions of the four outbreaks. (a) 2013. (b) 2013-2014. (c) 2015-2016. (d) 2016-2017.

**Figure 3:**
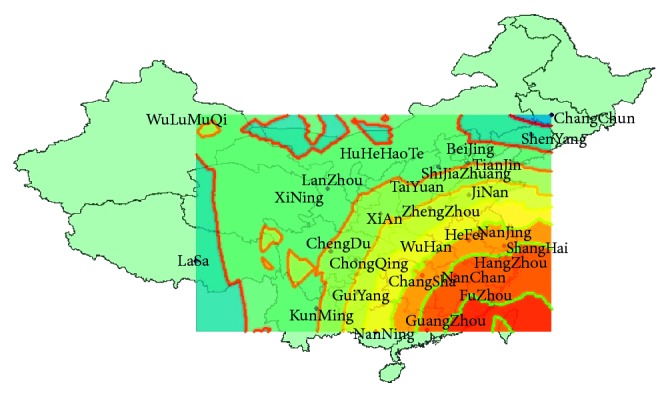
The distribution of all infections as of May 2017.

It seems that the A H7N9 influenza virus has spread to most provinces of China through the case reports. In purpose of understanding the range expansion, we will explore the spatial distributions of human infections in mainland China. By the method of GIS (Geographic Information Systems) [18, 19], based on the spatial data of the locations of the cases, using the module of geostatistical analyst—ArcGIS (a platform, which provides tools for mapping and spatial reasoning), we get the spatial distribution maps of human infections in different waves based on the ordinary kriging interpolation [20, 21], a spatial interpolation methodology that can compensate the lack of data and is an exact and unbiased interpolator. In Figure 2, we can get that the first wave of human infections with the A H7N9 avian influenza virus is mainly in the Yangtze River Delta area; the highest incidence regions are Shanghai, Jiangsu, and Zhejiang and diminish to the periphery radially. From October 2013 to May 2014, the center of the infections began to move southward, in addition to the Yangtze River Delta area (which points to the regions including Shanghai, Zhejiang province, Jiangsu province, and Anhui province), Guangdong has become the subcenter belt with a high incidence. From October 2015 to May 2016, the higher incidence area is still the southeast coastal area and less cases in inland areas but the geographical distribution of the epidemic has clearly expanded. From October 2016 to May 2017, infection areas are expanded widely. In fact, from reports, we know that in many areas the first case of human infection with the A H7N9 emerged, such as Gansu, Shanxi, and Inner Mongolia Autonomous Region. The first confirmed case was reported in April 2017 in Gansu, in May the first case in Shanxi was reported, and in May 31st in Inner Mongolia Autonomous Region, the first case was reported. At this point, cases of human infections with the A H7N9 avian influenza virus are present in most of mainland China.  Figure 3 is the distribution of all infections as of May 2017.

### 2.1. Remarks

1. The ordinary kriging interpolation effect of the third wave is similar to that of the second one and the graph is omitted here.
2. Because there are only few occasional cases during June to September, we define October 1 to May 31 of the second year as a wave of the outbreak. Although the WHO defines October 1 to September 30 of the second year as a wave of the epidemic, it does not affect the research results. Because in these five months, from June to September, there are 2 cases in 2013, 5 cases in 2014, 2 cases in 2015, 5 cases in 2016, and 6 cases in 2017.
3. All data are collected from the WHO and China CDC.
4. Distribution maps with data identifications are shown in Appendix.

## 3. Model Establishment

The spatial distributions suggest that the virus has spread. But, most of the new region cases do not involve the intercity movements, and there is no intercity live poultry trade in most areas in mainland China. We believe that the main medium of transmission is the virus in air. Therefore, we will formulate a model including the virus in air. Of course, it is more reasonable to consider the network model where the nodes are singe dynamic models and the links are intercity transportation if the intercity live trade is ubiquitous. And, if the human-to-human spread is confirmed, it is also reasonable to create a network model where the nodes are people and the links are the contacts among people. Thence, in the following, we turn to study the spread dynamically by establishing a mathematical model with a class of the virus in the environment.

Incorporating the virus in environment into the model is also based on the following facts. China CDC had deemed that 20 percent poultry in the LPMs carried the virus which is almost completely homologous to that in the human infection cases, and the homology reaches to more than 99.4 percent. It is very likely that human infections with avian influenza viruses can happen when a person touches something that has virus on it then touches his mouth, eyes, or nose. But, an undeniable fact is that only a very small number of human cases have been identified of the direct contact with poultry or the utensils; most cases simply stated that they had been to the LPMs. And some infections even have been identified that they had not contacted with live poultry or LPMs. That is to say, it seems that human infections with the A H7N9 virus can happen when virus is in the air (in droplets or possibly dust) and a person breathes it in. The spatial distributions illustrate the expansion which strengthens us that the virus in air can cause human infections. Many investigations had verified that the positive rates of H5, H7, and H9 avian influenza viruses were authentic in the out-environment where the poultry is sold or slaughtered. For instance, Chen et al. [22] gave the result that a significant higher positive sample rate was found in environmental samples for the reason that 44.4 percent of retail LPMs and 50.0 percent of wholesale LPMs were confirmed to be contaminated. He et al. [23] pointed out that H7N9 virus was detected in the environment and positive detection of H7N9 virus during environment surveillance increased from the first to the third wave. Ye et al. [24] had deduced conclusions that avian influenza A virus subtype H5, H7, and H9 circulate in the environment. That is, the environment was contaminated with avian influenza A H7N9 virus, and the risk of human infection exists in the environment. Therefore, we should incorporate the viruses that exist in the environment into the model when investigating the spread of A H7N9 avian influenza virus. The virus in the environment is denoted by *V*(*t*). Suppose the viruses in the environment are shed from virus-carried poultry, the shedding rate is *ε*. *p* > 0 shows the natural decay rate of virus in the environment and *q* > 0 represents the reduction rate due to people's disinfection measures.

The densities of susceptible and infected poultry population are denoted by *S*_*a*_(*t*) and *I*_*a*_(*t*), respectively. Because the A H7N9 avian influenza virus does not cause clinical signs in the infected poultry or all the identified infected poultry can be killed and buried due to easy identification, we modify the infected poultry as *E*_*a*_(*t*) (not *I*_*a*_(*t*)), also called as the virus-carried poultry. It is well known that the recruitment is prerequisite in the poultry industry. Let *A* > 0 be the constant recruitment rate of poultry, including the susceptible and the virus-carried poultry population. 0 ≤ *a* ≤ 1 is the proportion of the virus-carried poultry in the recruitment. And, mobility has been the symbol of modern life. Let *B* > 0 be the constant recruitment rate of people in the outbreak region. *d* > 0 is the natural death rate of the poultry population. *ρ* > 0 is the natural death rate of humans.

Suppose the virus-carried poultry is generated through infection of the susceptible poultry, the general incidence function *β*(*N*_1_)*S*_*a*_*E*_*a*_, *N*_1_=*S*_*a*_+*E*_*a*_, which satisfies the conditions [25]

(1) $$\begin{array}{c} \beta \left(N_{1}\right)>0,\beta^{\prime }\left(N_{1}\right)\leq 0,{\left[\beta \left(N_{1}\right)N_{1}\right]}^{\prime }\geq 0,\\ {\left[\beta^{\prime }\left(N_{1}\right)\right]}^{2}+{\left[\beta \left(N_{1}\right)N_{1}\right]}^{2}>0, \end{array}$$

is used to describe the contagion behaviour in the poultry for the reason that poultry population is large and it is densely populated.

It is known that for the instinct self-protection of humans, with the increase of human infection cases, authorities concerned would launch awareness campaigns to make people recognize the dire consequence caused by the avian influenza virus, and then the public get disease-related information and various protective measures via authorities and mass media. As a result, most people reduce their exposure to poultry and the related out-environment, and then the incidence will decrease. For the reason that the general nonmonotonic functions *g*_*i*_(*x*)(*i*=1,2) are used to characterize human infection rate, where *g*_*i*_(*x*) satisfy *g*_*i*_(0)=0 and *g*_*i*_′(0^+^) > 0 and that *g*_*i*_(*x*) will decrease when *x* is large relatively, Xiao and Rui [26] used specific functions ((kIS)/(1+*αI*^2^)) to express the psychosocial effect. Xiang et al. [27], Mukandavire et al. [28], and Bhunu et al. [29] used linear proportions to characterize the social psychology effect in their drink dynamics and HIV models, respectively.

People in the outbreak region are classified as the susceptible, the infected, and the recovered classes, denoted by *S*_*h*_(*t*), *I*_*h*_(*t*) and *R*_*h*_(*t*), respectively. The A H7N9 avian influenza virus causes severe death in human infection cases; *δ* > 0 is the additional death rate caused by the disease. *γ* > 0 is the recovery rate owing to the medical treatments.

**Figure 4:**
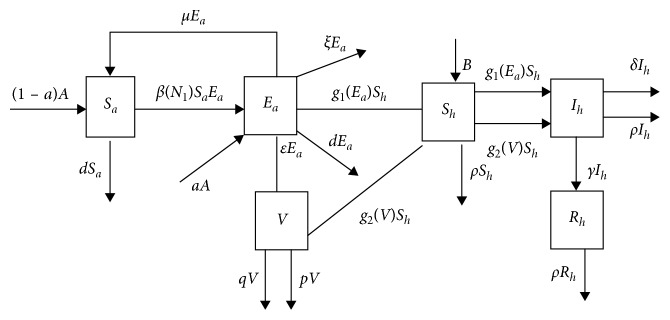
The spread mechanism.

Summing up, the spread mechanism is presented in Figure 4. And we formulate an SEV-SIR avian-human influenza model as follows:

(2) $$\begin{array}{c} \left\{\begin{array}{c} \frac{dS_{a}}{dt}=\left(1-a\right)A-\beta \left(N_{1}\right)S_{a}E_{a}-dS_{a}+\mu E_{a},\\ \frac{dE_{a}}{dt}=aA+\beta \left(N_{1}\right)S_{a}E_{a}-dE_{a}-\xi E_{a}-\mu E_{a},\\ \frac{dV}{dt}=\varepsilon E_{a}-pV-qV,\\ \frac{dS_{h}}{dt}=B-g_{1}\left(E_{a}\right)S_{h}-g_{2}\left(V\right)S_{h}-\rho S_{h},\\ \frac{dI_{h}}{dt}=g_{1}\left(E_{a}\right)S_{h}+g_{2}\left(V\right)S_{h}-\delta I_{h}-\rho I_{h}-\gamma I_{h},\\ \frac{dR_{h}}{dt}=\gamma I_{h}-\rho R_{h}, \end{array}\right. \end{array}$$

with the initial conditions *S*_*a*_(0)=*S*_*a*_0__ > 0, *E*_*a*_(0)=*E*_*a*_0__ > 0, *V*(0)=*V*_0_ ≥ 0, *S*_*h*_(0)=*S*_*h*_0__ > 0, *I*_*h*_(0)=*I*_*h*_0__ ≥ 0, *R*_*h*_(0)=*R*_*h*_0__ ≥ 0.

In this model, for the case of human infections with the low pathogenic avian influenza virus, *ξ* expresses the extra death rate due to the carried virus which weakens the health of poultry. In fact, entering into 2017, the new H7N9 virus had evolved into a highly pathogenic virus. *ξ* also expresses the incidence rate. And because the diseased individual is easy to be identified and people will kill all the diseased poultry, the latency actually spreads the disease. Therefore, the above model is still suitable to describe the actual spread for the case of human infections with the highly pathogenic avian influenza virus. That is, the model can characterize the dynamics of human infections with both the highly pathogenic avian influenza (HPAI) virus and the low pathogenic avian influenza (LPAI) virus. *μ* denotes the possible self-healing rate or the cure rate in poultry owing to the active treatment of people.

It is obvious that all the solutions initiating in *ℝ*_+_^6^ exist continuously for all *t* ≥ 0 and are unique. Where *ℝ*_+_^6^={(*x*, *y*, *z*, *u*, *v*, *w*) ∈ *ℝ*^6^ : *x* ≥ 0, *y* ≥ 0, *z* ≥ 0, *u* ≥ 0, *v* ≥ 0, *w* ≥ 0}.

## 4. Dynamic Analysis

### 4.1. Biological Validity

For the biological reality, we should prove the boundedness of the solutions of system (2) firstly.

**Theorem 1 .**

All the solutions of system (2) are ultimately uniform bounded and system (2) is dissipative.

**Proof**

(*S*_*a*_(*t*), *E*_*a*_(*t*), *V*(*t*), *S*_*h*_(*t*), *I*_*h*_(*t*), *R*_*h*_(*t*)) is any solution with the initial conditions *S*_*a*_0__ > 0, *E*_*a*_0__ ≥ 0, *V*_0_ ≥ 0, *S*_*h*_0__ > 0, *I*_*h*_0__ ≥ 0, *R*_*h*_0__ ≥ 0. Let *N*_1_(*t*)=*S*_*a*_(*t*)+*E*_*a*_(*t*), *N*_2_(*t*)=*S*_*h*_(*t*)+*I*_*h*_(*t*)+*R*_*h*_(*t*), *W*(*t*)=*N*_1_(*t*)+*V*(*t*)+*N*_2_(*t*), *ω*_1_=*d*+*ξ*+*μ*, *ω*_2_=*p*+*q*, *ω*_3_=*ρ*+*δ*+*γ*; then,

(3) $$\begin{array}{c} \frac{dN_{1}}{dt}=A-dN_{1}-\xi E_{a}\\ \leq A-dN_{1}. \end{array}$$

By the differential inequality, we obtain

(4) $$\begin{array}{c} 0\leq N_{1}\left(t\right)\leq \frac{A}{d}+N_{1}\left(S_{a_{0}},E_{a_{0}}\right)\cdot e^{-dt}, \end{array}$$

which implies *N*_1_ ≤ (*A*/*d*) as *t*⟶+*∞*. Similarly, we have

(5) $$\begin{array}{c} \frac{dN_{1}}{dt}\geq A-\left(d+\xi \right)N_{1}, \end{array}$$

and *N*_1_ ≥ (*A*/(*d*+*ξ*)) as *t*⟶+*∞*. In the same way, 0 ≤ *V* ≤ ((*Aε*)/(*dω*_2_)) and (*B*/(*δ*+*ρ*)) ≤ *N*_2_ ≤ (*B*/*ρ*) as *t*⟶+*∞*. And

(6) $$\begin{array}{c} \frac{dW}{dt}=A+B-dN_{1}-\xi E_{a}+\varepsilon E_{a}-\omega_{2}V-\rho N_{2}-\delta I_{h}\\ \leq A+B+\frac{\varepsilon A}{d}-\theta W, \end{array}$$

where *θ*=min{*d*, *ω*_2_, *ρ*}. Then, we obtain

(7) $$\begin{array}{c} 0\leq W\left(t\right)\leq \frac{A+B+\varepsilon A/d}{\theta }+W\left(S_{a_{0}},E_{a_{0}},V_{0},S_{h_{0}},I_{h_{0}},R_{h_{0}}\right)\cdot e^{-\theta t}, \end{array}$$

which implies *W* ≤ ((*A*+*B*+*εA*/*d*)/*θ*) as *t*⟶+*∞*. Similarly, we have

(8) $$\begin{array}{c} \frac{dW}{dt}\geq A+B-\left(d+\xi +\omega_{2}+\delta +\rho \right)W,\\ W\geq \frac{A+B}{d+\xi +\omega_{2}+\delta +\rho }\text{,}\;\;t⟶+\infty . \end{array}$$

Therefore, all the solutions of system (2) that initiate in *R*_+_^6^ are confined in the following region for sufficiently large time:

(9) $$\begin{array}{c} \Omega =\left\{\left(S_{a},E_{a},V,S_{h},I_{h},R_{h}\right)\in R_{+}^{6}\text{:}\frac{A+B}{d+\xi +\omega_{2}+\delta +\rho }\leq W<\frac{A+B+\varepsilon A/d}{\theta },\frac{A}{d+\xi }\leq N_{1}\leq \frac{A}{d},0<V\leq \frac{A\varepsilon }{d\omega_{2}},\frac{B}{\delta +\rho }\leq N_{2}\leq \frac{B}{\rho }\right\}. \end{array}$$

Hence, the theorem holds.

### 4.2. Existence of Equilibria

In the need of investigating the dynamic behaviours of the model, we firstly analyze the existence of equilibrium point. Obviously, it depends on the following algebra equations:

(10) $$\begin{array}{c} \left\{\begin{array}{c} A-dN_{1}-\xi E_{a}=0,\\ aA+\beta \left(N_{1}\right)\left(N_{1}-E_{a}\right)E_{a}=\omega_{1}E_{a}. \end{array}\right. \end{array}$$

By the direct calculation, we have the following:

(i) If *a*=0 and *ξ* ≠ 0, it is easy that there is a disease-free equilibrium point *M*_0_((*A*/*d*), 0,0, (*B*/*ρ*), 0,0) and a positive equilibrium point *M*^*∗*^(*S*_*a*_^*∗*^, *E*_*a*_^*∗*^, *V*^*∗*^, *S*_*h*_^*∗*^, *I*_*h*_^*∗*^, *R*_*h*_^*∗*^) if and only if (*A*/*d*)*β*(*A*/*d*) > *ω*_1_, where *S*_*a*_^*∗*^=(*ω*_1_/(*β*(*N*_1_^*∗*^))), *E*_*a*_^*∗*^=((*A* − *dN*_1_^*∗*^)/*ξ*), *V*^*∗*^=(*εE*_*a*_^*∗*^/*ω*_2_), *S*_*h*_^*∗*^=(*B*/(*g*_1_(*E*_*a*_^*∗*^)+*g*_2_(*V*^*∗*^)+*ω*_3_)), *I*_*h*_^*∗*^=(((*g*_1_(*E*_*a*_^*∗*^)+*g*_2_(*V*^*∗*^))*S*_*h*_^*∗*^)/*ω*_3_), *R*_*h*_^*∗*^=(*γI*_*h*_^*∗*^/*ρ*). *N*_1_^*∗*^ ∈ (0, (*A*/*d*)) is the unique positive root of equation
  (11) $$\begin{array}{c} \beta \left(N_{1}\right)N_{1}-\beta \left(N_{1}\right)\frac{A-dN_{1}}{\xi }-\omega_{1}=0. \end{array}$$

In fact, denote *F*(*N*_1_)=*β*(*N*_1_)*N*_1_ − *β*(*N*_1_)((*A* − *dN*_1_)/*ξ*) − *ω*_1_, there is *F*(0^+^)=−*ω*_1_ < 0, *F*(*A*/*d*)^−^=*β*(*A*/*d*)(*A*/*d*) − *ω*_1_ and *F*′(*N*_1_)=(1+(*d*/*ξ*))[*β*(*N*_1_)*N*_1_]′ − *β*′(*N*_1_)(*A*/*ξ*) > 0.

(ii) If *a*=0 and *ξ*=0, the disease-free equilibrium point *M*_0_((*A*/*d*), 0,0, (*B*/*ρ*), 0,0) exists. And directly there is a unique *E*_*a*_^*∗*^=(*A*/*d*) − ((*d*+*μ*)/(*β*(*A*/*d*))) ∈ (0, (*A*/*d*)) if and only if (*A*/*d*)*β*(*A*/*d*) > (*d*+*μ*), corresponding to a positive equilibrium point *M*^*∗*^, where *S*_*a*_^*∗*^=((*d*+*μ*)/*β*(*A*/*d*)).
(iii) If *a* ≠ 0 and *ξ*=0, we have
  (12) $$\begin{array}{c} -\beta \left(\frac{A}{d}\right)E_{\text{a}}^{2}+\left[\beta \left(\frac{A}{d}\right)\frac{A}{d}-\omega_{1}\right]E_{\text{a}}+aA=0. \end{array}$$

Denote the left of equation (12) as *f*(*E*_*a*_); then, *f*(0^+^)=*aA* > 0 and *f*(*A*/*d*)^−^=(*A*/*d*)[(*a* − 1)*d* − *ξ* − *μ*] < 0. That is, equation (12) has a unique positive solution *E*_*a*_^*∗*^ ∈ (0, (*A*/*d*)) by the property of quadratic function. Then, *S*_*a*_^*∗*^=(*A*/*d*) − *E*_*a*_^*∗*^. In other words, system (2) has a unique positive equilibrium point *M*^*∗*^ in Ω.

(iv) If *a* ≠ 0 and *ξ* ≠ 0, we have
  (13) $$\begin{array}{c} \frac{\xi aA}{A-dN_{1}}+\beta \left(N_{1}\right)N_{1}-\beta \left(N_{1}\right)\frac{A-dN_{1}}{\xi }-\omega_{1}=0. \end{array}$$

Let *h*(*N*_1_)=((*ξaA*)/(*A* − *dN*_1_))+*β*(*N*_1_)*N*_1_ − *β*(*N*_1_)((*A* − *dN*_1_)/*ξ*) − *ω*_1_. Then, *h*(0^+^)=*ξ*(*a* − 1) − ((*β*(0^+^)*A*)/*ξ*) − *d* − *μ* < 0, *h*(*A*/*d*)^+^⟶+*∞*, and *h*′(*N*_1_)=((*ξadA*)/(*A* − *dN*_1_)^2^)+[*β*(*N*_1_)*N*_1_]′ − ((*Aβ*′(*N*_1_))/*ξ*) > 0. That is, there is a *N*_1_^*∗*^ ∈ (0, (*A*/*d*)) which is the unique positive root of equation (13). Then, *E*_*a*_^*∗*^=((*A* − *dN*_1_^*∗*^)/*ξ*) and *S*_*a*_^*∗*^=*N*_1_^*∗*^ − *E*_*a*_^*∗*^.

The expressions of *V*^*∗*^, *S*_*h*_^*∗*^, *I*_*h*_^*∗*^ and *R*_*h*_^*∗*^ in (ii), (iii), and (iv) are still those in (i). Therefore, we have the following conclusions:

**Theorem 2 .**

(i) If *a* ≠ 0, there is a unique positive equilibrium point *M*^*∗*^; (ii) If *a*=0, there is a disease-free equilibrium point *M*_0_, and a positive equilibrium point *M*^*∗*^ also exists if and only if (*A*/*d*)*β*(*A*/*d*) > *ω*_1_.

*Remark.* There are different coordinate representations of the positive equilibrium point *M*^*∗*^ in the cases of *ξ* ≠ 0 and *ξ*=0. That is, *M*^*∗*^ is just a symbol of the positive equilibrium point.

In view of the dynamics of infectious disease, the existence of a positive equilibrium point means the epidemic may develop into endemic disease. Correspondingly, the existence of the disease-free equilibrium point means the epidemic may be eliminated. The parameter *a*=0 means the latency in the recruitment can be excluded absolutely. Therefore, the theorem indicates that the first control measure we try to do is to develop technology and equipment in purpose of prohibiting the latency in input. Furthermore, if we can control the size of input and the spread of virus in poultry or if we can strengthen the preventive treatment in poultry, it is expected to eliminate the epidemic. If we could not curb the existence of the latency in input, the outbreak may be an endemic disease.

### 4.3. Stability of Equilibria

The stability of an equilibrium point reflects the dynamic behaviour of a system. In the following, we investigate an equivalent system:

(14) $$\begin{array}{c} \left\{\begin{array}{c} \frac{dN_{1}}{dt}=A-dN_{1}-\xi E_{a},\\ \frac{dE_{a}}{dt}=aA+\beta \left(N_{1}\right)\left(N_{1}-E_{a}\right)E_{a}-\omega_{1}E_{a},\\ \frac{dV}{dt}=\varepsilon E_{a}-\omega_{2}V,\\ \frac{dS_{h}}{dt}=B-g_{1}\left(E_{a}\right)S_{h}-g_{2}\left(V\right)S_{h}-\rho S_{h},\\ \frac{dI_{h}}{dt}=g_{1}\left(E_{a}\right)S_{h}+g_{2}\left(V\right)S_{h}-\omega_{3}I_{h},\\ \frac{dR_{h}}{dt}=\gamma I_{h}-\rho R_{h}. \end{array}\right. \end{array}$$

The Jacobian matrix of system (14) is given as

(15) $$\begin{array}{c} J=\left(\begin{array}{cc} C & O\\ L & D \end{array}\right), \end{array}$$

where

(16) $$\begin{array}{c} C=\left(\begin{array}{ccc} -d & -\xi & 0\\ {\left(\beta \left(N_{1}\right)N_{1}\right)}^{\prime }E_{a}-\beta^{\prime }\left(N_{1}\right)E_{a}^{2} & \beta \left(N_{1}\right)N_{1}-2\beta \left(N_{1}\right)E_{a}-\omega_{1} & 0\\ 0 & \varepsilon & -\omega_{2} \end{array}\right),\\ D=\left(\begin{array}{ccc} -g_{1}\left(E_{a}\right)-g_{2}\left(V\right)-\rho & 0 & 0\\ g_{1}\left(E_{a}\right)+g_{2}\left(V\right) & -\omega_{3} & 0\\ 0 & \gamma & -\rho \end{array}\right). \end{array}$$

Therefore, *J* evaluated at an equilibrium point is stable if and only if so are *C* and *D*. Obviously, all the eigenvalues of the submatrix *D* have the negative real parts; then, the local stability of an equilibrium point depends on the evaluation of submatrix *C*. The Jacobian matrix *C* corresponding to the source-subsystem (17) is as follows:

(17) $$\begin{array}{c} \left\{\begin{array}{c} \frac{dN_{1}}{dt}=A-dN_{1}-\xi E_{a},\\ \frac{dE_{a}}{dt}=aA+\beta \left(N_{1}\right)\left(N_{1}-E_{a}\right)E_{a}-\omega_{1}E_{a},\\ \frac{dV}{dt}=\varepsilon E_{a}-\omega_{2}V. \end{array}\right. \end{array}$$

The Jacobian matrix *C* evaluated at equilibrium point *M*_0_ and *M*^*∗*^ is as follows:

(18) $$\begin{array}{c} J;C\left|\;\right.{}_{M_{0}}=\left(\begin{array}{ccc} -d & -\xi & 0\\ 0 & \beta \left(\frac{A}{d}\right)\frac{A}{d}-\omega_{1} & 0\\ 0 & \varepsilon & -\omega_{2} \end{array}\right),\\ J;C\left|\;\right.{}_{M^{*}}=\left(\begin{array}{ccc} -d & -\xi & 0\\ -\wedge_{2} & -\wedge_{1}-\beta \left(N_{1}^{*}\right)E_{a}^{*} & 0\\ 0 & \varepsilon & -\omega_{2} \end{array}\right), \end{array}$$

where

(19) $$\begin{array}{c} \wedge_{1}=\omega_{1}-\beta \left(N_{1}^{*}\right)N_{1}^{*}+\beta \left(N_{1}^{*}\right)E_{a}^{*},\\ \wedge_{2}=-{\left[\beta \left(N_{1}^{*}\right)N_{1}^{*}\right]}^{\prime }E_{a}^{*}+\beta^{\prime }\left(N_{1}^{*}\right){\left(E_{a}^{*}\right)}^{2}. \end{array}$$

Obviously, if (*A*/*d*)*β*(*A*/*d*) < *ω*_1_, *M*_0_ is locally asymptotically stable (LAS).

The eigenvalues corresponding to *M*^*∗*^ are given by *λ*=−*ω*_2_ < 0 and the roots of following equation:

(20) $$\begin{array}{c} \lambda^{2}+\left[d+\beta \left(N_{1}^{*}\right)E_{a}^{*}+\wedge_{1}\right]\lambda +d\left[\wedge_{1}+\beta \left(N_{1}^{*}\right)E_{a}^{*}\right]-\wedge_{2}\xi =0. \end{array}$$

At *M*^*∗*^, it is satisfied that ∧_1_=*ω*_1_ − *β*(*N*_1_^*∗*^)*N*_1_^*∗*^+*β*(*N*_1_^*∗*^)*E*_*a*_^*∗*^=(*aA*/*E*_*a*_^*∗*^) > 0. And ∧_2_ < 0 because of *β*′(*N*_1_) ≤ 0 and [*β*(*N*_1_)*N*_1_]′ ≥ 0. Therefore, for the two roots of equation (20), there is *λ*_1_+*λ*_2_=−(*d*+*β*(*N*_1_^*∗*^)*E*_*a*_^*∗*^+∧_1_) < 0 and *λ*_1_*λ*_2_=*d*(∧_1_+*β*(*N*_1_^*∗*^)*E*_*a*_^*∗*^) − ∧_2_*ζ* > 0.

Summing up, we have the following conclusions.

**Theorem 3 .**

(i) If (*A*/*d*)*β*(*A*/*d*) < *ω*_1_, *M*_0_ is locally asymptotically stable; (ii) *M*^*∗*^ is locally asymptotically stable if it exists.

Next, we study the global asymptotically stability (GAS) of equilibria. Notice that the source-subsystem (17) is independent of the human-subsystem, we investigate the source-subsystem (17) firstly. When investigating the GAS of *M*_0_, there is *a*=0 in equation (17). Denote *M*_0_*a*__ as the corresponding point of *M*_0_ for the source-subsystem (17). We choose the Lyapunov function *L*=*E*_*a*_. With *β*(*N*_1_) > 0 and [*β*(*N*_1_)*N*_1_]′ ≥ 0, we have

(21) $$\begin{array}{c} \dot{L}\left|{}_{\left(5\right)}\right.=\beta \left(N_{1}\right)\left(N_{1}-E_{a}\right)E_{a}-\omega_{1}E_{a}\leq \left[\beta \left(N_{1}\right)N_{1}-\omega_{1}\right]E_{a}\leq \left[\beta \left(\frac{A}{d}\right)\frac{A}{d}-\omega_{1}\right]E_{a}. \end{array}$$

Then $\dot{L}\left|\left(5\right)\right.\leq 0$ as *β*(*A*/*d*)(*A*/*d*) ≤ *ω*_1_. Let $G=\left\{\left(N_{1},E_{a},V\right)\in {\mathbb{R}}_{+}^{3}:\dot{L}=0\right\}=\left\{E_{a}=0\right\}$. That is, all the solutions of the source-subsystem (17) will approach the *N*_1_ − *V* plane based on the LaSalle invariance principle.

Furthermore, it is easy that *N*_1_⟶(*A*/*d*) and *V*⟶0 as *E*_*a*_⟶0 by equation (17). Therefore, *M*_0_*a*__((*A*/*d*), 0,0) is GAS as (*A*/*d*)*β*(*A*/*d*) ≤ *ω*_1_. Again, it is obvious that *S*_*h*_⟶(*B*/*V*), *I*_*h*_⟶0 and *R*_*h*_⟶0 as *t*⟶+*∞* by the theory of limit system.

**Theorem 4 .**

If (*A*/*d*)*β*(*A*/*d*) < *ω*_1_, *M*_0_ is globally asymptotically stable.

In what follows, we discuss the stability of *M*^*∗*^ when it exists. For the equivalent source-subsystem:

(22) $$\begin{array}{c} \left\{\begin{array}{c} \frac{dS_{a}}{dt}=\left(1-a\right)A-\beta \left(N_{1}\right)S_{a}E_{a}-dS_{a}+\mu E_{a},\\ \frac{dE_{a}}{dt}=aA+\beta \left(N_{1}\right)S_{a}E_{a}-dE_{a}-\xi E_{a}-\mu E_{a},\\ \frac{dV}{dt}=\varepsilon E_{a}-pV-qV. \end{array}\right. \end{array}$$

Correspondingly, all the solutions of system (22) are confined in the region

(23) $$\begin{array}{c} \Omega_{a}=\left\{\left(S_{a},E_{a},V\right)\in R_{+}^{3}:\;\;\frac{A}{d+\xi }\leq S_{a}+E_{a}+V\leq \frac{A}{d},0<V\leq \frac{A\varepsilon }{d\omega_{2}}\right\}, \end{array}$$

for sufficiently large time and system (22) is dissipative. It is clear that Ω_*a*_ is a compact subset of *ℝ*_+_^3^ and is forward invariant. And it is easy that the maximal invariant set on the ∂Ω_*a*_ is the singleton *M*_0_*a*__ and it is isolated. By the theorem in [30], system (7) is uniformly persistent if and only if *M*_0_*a*__ is unstable. While, *M*_0_*a*__ is an unstable saddle point as (*A*/*d*)*β*(*A*/*d*) > *ω*_1_.

For *a* > 0, define that

(24) $$\begin{array}{c} D_{+}=\left\{\left(S_{a},E_{a},V\right)\in R_{+}^{3}:k\leq N_{1}+V\leq K\right\},\\ D_{S_{a}}=\left\{\left(S_{a},E_{a},V\right)\in R_{+}^{3}:S_{a}=0,k\leq N_{1}+V\leq K\right\},\\ D_{E_{a}}=\left\{\left(S_{a},E_{a},V\right)\in R_{+}^{3}:S_{a}\geq k_{S_{a}},E_{a}=0,k\leq N_{1}+V\leq K\right\},\\ D_{V}=\left\{\left(S_{a},E_{a},V\right)\in R_{+}^{3}:S_{a}\geq k_{S_{a}},E_{a}\geq k_{E_{a}},V=0,k\leq N_{1}+V\leq K\right\}, \end{array}$$

where *k*_*i*_ > 0, *i*=*S*_*a*_, *E*_*a*_, *V*. The existence of *k* > 0 and *K* > 0 are due to the results of Theorem 4.1. It is obvious that *D*_*S*_*a*__ is a compact subset of *D*_+_. Let *V*=*S*_*a*_, then *V* : *D*_+_⟶*ℝ*_+_ is *C*^1^ and satisfies *V*(*ζ*)=0 if and only if *ζ* ∈ *D*_*S*_*a*__. It is clear that $\dot{V}\left(\zeta \right)=\left(1-a\right)A+\mu E_{a}>0$ for any *ζ* ∈ *D*_*S*_*a*__. Therefore, by the Lyapunov instability theorem, there exists *k*_*S*_*a*__ > 0 such that lim inf_*t*⟶+*∞*_*S*_*a*_(*t*) ≥ *k*_*S*_*a*__ for any *ψ*_0_ ∈ *D*_+_/*D*_*S*_*a*__ (*ψ*_0_ is the initial value). Similarly, we have lim inf_*t*⟶+*∞*_*E*_*a*_(*t*) ≥ *k*_*E*_*a*__, lim inf_*t*⟶+*∞*_*V*(*t*) ≥ *k*_*V*_ for *k*_*E*_*a*__ > 0, *k*_*V*_ > 0. It means that any positive solution of system (22) is repelled uniformly from all the boundary planes [31]. Together with the boundedness from Theorem 4.1, we obtain that system (22) is uniformly persistent [30]. *M*_*a*_^*∗*^ is the corresponding point of *M*^*∗*^ for the subsystem (22). Therefore, we have the following conclusion.

**Theorem 5 .**

System (22) is uniformly persistent if *M*_*a*_^*∗*^ exists.

Next, we will prove the globally asymptotical stability of *M*^*∗*^. At first, we introduce a definition of the second additive compound matrix and three lemmas.

Second additive compound matrix *A*^[2]^: let *A* be a linear operator on **R**^*n*^ and also denote its matrix representation with respect to the standard basis of **R**^*n*^. *A* induces canonically a linear operator *A*^[2]^ on ∧^2^**R**^*n*^: for *u*_1_, *u*_2_ ∈ **R**^*n*^, define *A*^[2]^(*u*_1_∧*u*_2_)=*A*(*u*_1_)∧*u*_2_+*u*_1_∧*A*(*u*_2_) and extend the definition over ∧^2^**R**^*n*^ by linearity. This is an $\left(\begin{array}{c} n\\ 2 \end{array}\right)\times \left(\begin{array}{c} n\\ 2 \end{array}\right)$ matrix with each entry as a linear expression of those of *A*. When *n*=3, *A*=(*a*_*ij*_); then,

(25) $$\begin{array}{c} A^{\left[2\right]}=\left(\begin{array}{ccc} a_{11}+a_{22} & a_{23} & -a_{13}\\ a_{32} & a_{11}+a_{33} & a_{12}\\ -a_{31} & a_{21} & a_{22}+a_{33} \end{array}\right). \end{array}$$

Detailed information of *A*^[2]^ refers to Fiedler [32] and Muldowney [33].

Let *x* ↦ *f*(*x*) ∈ **R**^*n*^ be a *C*^1^ function for *x* in an open set Ω⊂**R**^*n*^. Consider the differential equation

(26) $$\begin{array}{c} x^{\prime }=f\left(x\right). \end{array}$$

Denote *x*(*t*, *x*_0_) as the solution of equation (26) with respect to *x*(0, *x*_0_)=*x*_0_.

**Lemma 1 .**

(Li and Wang [34]). Assume that following conditions are satisfied: (1) there exists a compact absorbing set *K* ⊂ *D*; (2) there exists a unique equilibrium point $\bar{x}$ in *D*; (3) system *x*′=*f*(*x*) satisfies the property of Poincaré–Bendixson theorem; (4) for any periodic solution *x*=*x*(*t*) of the system with *x*(0) ∈ *D*, the corresponding second compound system *z*′(*t*)=((∂*f*^[2]^)/∂*x*)(*x*(*t*))*z*(*t*) is asymptotically stable; (5) ${\left(-1\right)}^{n}\text{det}\left(\left(\partial f/\partial x\right)\left(\bar{x}\right)\right)>0$.

Then, the unique equilibrium point $\bar{x}$ of the system is globally asymptotically stable in *D*.

**Lemma 2 .**

(Li and Wang [34]). For *n*=3 and *D* is convex, system *x*′=*f*(*x*) would meet the property of Poincaré–Bendixson theorem if it is a competitive system in *D*.

**Lemma 3 .**

(Bulter and Waltman [30]). Condition (1) of Lemma 4.1 is equivalent to the uniform persistence of the system if *D* is a bounded cone.

It is obvious Ω_*a*_ is a bounded cone, then condition (1) of Lemma 4.1 is satisfied by Theorem 4.5. Condition (2) of Lemma 4.1 is automatically satisfied. In the following, we will verify that system (22) is a competitive system. The Jacobian matrix of system (22) is

(27) $$\begin{array}{c} J_{1}=\left(\begin{array}{ccc} -\beta \left(N_{1}\right)S_{a}-\beta^{\prime }\left(N_{1}\right)S_{a}E_{a}-d & \mu -\beta \left(N_{1}\right)S_{a}-\beta^{\prime }\left(N_{1}\right)S_{a}E_{a} & 0\\ \beta \left(N_{1}\right)E_{a}+\beta^{\prime }\left(N_{1}\right)S_{a}E_{a} & \beta \left(N_{1}\right)S_{a}+\beta^{\prime }\left(N_{1}\right)S_{a}E_{a}-\omega_{1} & 0\\ 0 & \varepsilon & -\omega_{2} \end{array}\right). \end{array}$$

Let *H*=diag{1, −1,1}, then

(28) $$\begin{array}{c} HJ_{1}H=\left(\begin{array}{ccc} -\beta \left(N_{1}\right)S_{a}-\beta^{\prime }\left(N_{1}\right)S_{a}E_{a}-d & -\mu +\beta \left(N_{1}\right)S_{a}+\beta^{\prime }\left(N_{1}\right)S_{a}E_{a} & 0\\ -\beta \left(N_{1}\right)E_{a}-\beta^{\prime }\left(N_{1}\right)S_{a}E_{a} & \beta \left(N_{1}\right)S_{a}+\beta^{\prime }\left(N_{1}\right)S_{a}E_{a}-\omega_{1} & 0\\ 0 & -\varepsilon & -\omega_{2} \end{array}\right). \end{array}$$

Because

(29) $$\begin{array}{c} -\beta \left(N_{1}\right)E_{a}-\beta^{\prime }\left(N_{1}\right)S_{a}E_{a}=\left[-\beta \left(N_{1}\right)-\beta^{\prime }\left(N_{1}\right)N_{1}+\beta^{\prime }\left(N_{1}\right)E_{a}\right]E_{a}=\left\{-{\left[\beta \left(N_{1}\right)N_{1}\right]}^{\prime }+\beta^{\prime }\left(N_{1}\right)E_{a}\right\}E_{a}, \end{array}$$

and *β*′(*N*_1_) ≤ 0, [*β*(*N*_1_)*N*_1_]′ ≥ 0. Then, all the off-diagonal elements of *HJ*_1_*H* are not positive if *μ* ≥ *β*(*N*_1_)*S*_*a*_+*β*′(*N*_1_)*S*_*a*_*E*_*a*_. And ((∂*β*(*N*_1_)*S*_*a*_*E*_*a*_)/(∂*E*_*a*_))=*β*(*N*_1_)*S*_*a*_+*β*′(*N*_1_)*S*_*a*_*E*_*a*_. That is, system (22) is a competitive system if *μ* ≥ ((∂*β*(*N*_1_)*S*_*a*_*E*_*a*_)/(∂*E*_*a*_)).

Next, we will verify condition (4) of Lemma 4.1. Assume that *ϕ*(*t*)=(*S*_*a*_(*t*), *E*_*a*_(*t*), *V*(*t*)) is the nontrivial periodic solution of system (22) with (*S*_*a*_(0), *E*_*a*_(0), *V*(0)) ∈ Ω_*a*_, the minimum positive period *τ* > 0, and the trajectory {Γ(*t*):  0 ≤ *t* ≤ *τ*}. The second compound system *X*′(*t*)=(∂*f*^[2]^/∂*X*)*X*(*t*) of system (22)'s variational system *Y*′=*J*_1_*Y* at *ϕ*(*t*) is as follows:

(30) $$\begin{array}{c} \left\{\begin{array}{c} x^{\prime }=\left[\beta \left(N_{1}\right)S_{a}-\beta \left(N_{1}\right)E_{a}-d-\omega_{1}\right]x,\\ y^{\prime }=\varepsilon x+\left[-\beta^{\prime }\left(N_{1}\right)S_{a}E_{a}-\beta \left(N_{1}\right)E_{a}-d-\omega_{2}\right]y+\left[\mu -\beta \left(N_{1}\right)S_{a}-\beta^{\prime }\left(N_{1}\right)S_{a}E_{a}\right]z,\\ z^{\prime }=\left[\beta \left(N_{1}\right)E_{a}+\beta^{\prime }\left(N_{1}\right)S_{a}E_{a}\right]y+\left[\beta \left(N_{1}\right)S_{a}+\beta^{\prime }\left(N_{1}\right)S_{a}E_{a}-\omega_{1}-\omega_{2}\right]z. \end{array}\right. \end{array}$$

Let (*x*(*t*), *y*(*t*), *z*(*t*)) be a solution of *X*′(*t*)=((∂*f*^[2]^)/∂*X*)*X*(*t*); then,

(31) $$\begin{array}{c} D_{+}\left|x\left(t\right)\right|\leq \left[\beta \left(N_{1}\right)S_{a}-\beta \left(N_{1}\right)E_{a}-d-\omega_{1}\right]\left|x\left(t\right)\right|\leq \left[\beta \left(N_{1}\right)S_{a}-d-\omega_{1}\right]\left|x\left(t\right)\right|,\\ D_{+}\left|y\left(t\right)\right|\leq \varepsilon \left|x\left(t\right)\right|+\left[-\beta^{\prime }\left(N_{1}\right)S_{a}E_{a}-\beta \left(N_{1}\right)E_{a}-d-\omega_{2}\right]\left|y\left(t\right)\right|+\left[\mu -\beta \left(N_{1}\right)S_{a}-\beta^{\prime }\left(N_{1}\right)S_{a}E_{a}\right]\left|z\left(t\right)\right|,\\ D_{+}\left|z\left(t\right)\right|\leq \left[\beta \left(N_{1}\right)E_{a}+\beta^{\prime }\left(N_{1}\right)S_{a}E_{a}\right]\left|y\left(t\right)\right|+\left[\beta \left(N_{1}\right)S_{a}+\beta^{\prime }\left(N_{1}\right)S_{a}E_{a}-\omega_{1}-\omega_{2}\right]\left|z\left(t\right)\right|,\\ D_{+}\left(\left|y\left(t\right)\right|+\left|z\left(t\right)\right|\right)\leq \varepsilon \left|x\left(t\right)\right|-\left(d+\omega_{2}\right)\left|y\left(t\right)\right|+\left(\mu -\omega_{1}-\omega_{2}\right)\left|z\left(t\right)\right|\\ =\varepsilon \left|x\left(t\right)\right|-\left(d+\omega_{2}\right)\left|y\left(t\right)\right|-\left(d+\xi +\omega_{2}\right)\left|z\left(t\right)\right|\\ =\varepsilon \left|x\left(t\right)\right|-\left(d+\omega_{2}\right)\left(\left|y\left(t\right)\right|+\left|z\left(t\right)\right|\right)-\xi \left|z\left(t\right)\right|\\ \leq \varepsilon \left|x\left(t\right)\right|-\left(d+\omega_{2}\right)\left(\left|y\left(t\right)\right|+\left|z\left(t\right)\right|\right). \end{array}$$

Choosing the Lyapunov function, *V*(*x*, *y*, *z*, *S*_*a*_, *E*_*a*_, *v*)=sup{|*x*|, (*E*_*a*_/*v*)(|*y*|+|*z*|)}. By the uniform persistence, there is a certain distance between the periodic solution Γ(*t*)(*S*_*a*_(*t*), *E*_*a*_(*t*), *V*(*t*)) and the ∂Ω_*a*_. Therefore, ∃*c* > 0, such that

(32) $$\begin{array}{c} V\left(x,y,z,S_{a},E_{a},v\right)\geq c\text{sup}\left\{\left|x\right|,\left|y\right|,\left|z\right|\right\}, \end{array}$$

for all (*x*, *y*, *z*) ∈ *R*^3^ and (*S*_*a*_(*t*), *E*_*a*_(*t*), *V*(*t*)) ∈ Γ(*t*). Through direct calculation, we obtain that

(33) $$\begin{array}{c} D_{+}\frac{E_{a}}{v}\left(\left|y\left(t\right)\right|+\left|z\left(t\right)\right|\right)=\left(\frac{E_{a}^{\prime }}{E_{a}}-\frac{v^{\prime }}{v}\right)\left(\left|y\left(t\right)\right|+\left|z\left(t\right)\right|\right)+\frac{E_{a}}{v}D_{+}\left(\left|y\right|+\left|z\right|\right)\\ \leq \frac{\varepsilon E_{a}}{v}\left|x\left(t\right)\right|+\left(\frac{E_{a}^{\prime }}{E_{a}}-\frac{v^{\prime }}{v}-d-\omega_{2}\right)\frac{E_{a}}{v}\left(\left|y\left(t\right)\right|+\left|z\left(t\right)\right|\right). \end{array}$$

Then, *D*_+_|*V*(*t*)||≤max{*f*_1_(*t*), *f*_2_(*t*)}*V*(*t*), where

(34) $$\begin{array}{c} f_{1}\left(t\right)=\beta \left(N_{1}\right)S_{a}-d-\omega_{1},\\ f_{2}\left(t\right)=\frac{\varepsilon E_{a}}{v}+\frac{E_{a}^{\prime }}{E_{a}}-\frac{v^{\prime }}{v}-d-\omega_{2}. \end{array}$$

Via system (26), we have

(35) $$\begin{array}{c} \frac{E_{a}^{\prime }}{E_{a}}=\frac{aA}{E_{a}}+\beta \left(N_{1}\right)S_{a}-\omega_{1},\\ \frac{v^{\prime }}{v}=\frac{\varepsilon E_{a}}{v}-\omega_{2}. \end{array}$$

Then,

(36) $$\begin{array}{c} f_{1}\left(t\right)=\frac{E_{a}^{\prime }}{E_{a}}-\frac{aA}{E_{a}}-d,\\ f_{2}\left(t\right)=\frac{E_{a}^{\prime }}{E_{a}}-d. \end{array}$$

Therefore,

(37) $$\begin{array}{c} \text{max}\left\{f_{1}\left(t\right),f_{2}\left(t\right)\right\}\leq \frac{E_{a}^{\prime }}{E_{a}}-d,\\ {\int^{}}_{0}^{\tau }\text{max}\left\{f_{1}\left(t\right),f_{2}\left(t\right)\right\}dt\leq {\int^{}}_{0}^{\tau }\left(\frac{E_{a}^{\prime }}{E_{a}}-d\right)dt\\ =\ln E_{a}\left|\overset{\tau }{0}\right.-d\tau \\ =-d\tau . \end{array}$$

Thus, lim_*t*⟶+*∞*_*V*(*t*)=0. It means (*x*(*t*), *y*(*t*), *z*(*t*))⟶0 as *t*⟶+*∞*. That is, the second compound system *X*′(*t*)=(∂*f*^[2]^/∂*X*)*X*(*t*) is asymptotically stable.

Rewriting *J*_1_ as follows:

(38) $$\begin{array}{c} J_{1}=\left(\begin{array}{ccc} -\frac{\partial \beta \left(N_{1}\right)S_{a}E_{a}}{\partial S_{a}}-d & \mu -\frac{\partial \beta \left(N_{1}\right)S_{a}E_{a}}{\partial E_{a}} & 0\\ \frac{\partial \beta \left(N_{1}\right)S_{a}E_{a}}{\partial S_{a}} & \frac{\partial \beta \left(N_{1}\right)S_{a}E_{a}}{\partial E_{a}}-\omega_{1} & 0\\ 0 & \varepsilon & -\omega_{2} \end{array}\right). \end{array}$$

Then,

(39) $$\begin{array}{c} \text{det}\left(\frac{\partial f}{\partial x}\left(\bar{x}\right)\right)={\left.-\omega_{2}\left[\frac{\partial \beta \left(N_{1}\right)S_{a}E_{a}}{\partial S_{a}}\cdot \frac{\partial \beta \left(N_{1}\right)S_{a}E_{a}}{\partial E_{a}}+\omega_{1}\frac{\partial \beta \left(N_{1}\right)S_{a}E_{a}}{\partial S_{a}}-d\frac{\partial \beta \left(N_{1}\right)S_{a}E_{a}}{\partial E_{a}}+d\omega_{1}-\mu \frac{\partial \beta \left(N_{1}\right)S_{a}E_{a}}{\partial S_{a}}+\frac{\partial \beta \left(N_{1}\right)S_{a}E_{a}}{\partial S_{a}}\cdot \frac{\partial \beta \left(N_{1}\right)S_{a}E_{a}}{\partial E_{a}}\right]\right|}_{M_{a}^{*}}\\ ={\left.-\omega_{2}\left[d\left(\omega_{1}-\frac{\partial \beta \left(N_{1}\right)S_{a}E_{a}}{\partial E_{a}}\right)+\left(d+\zeta \right)\frac{\partial \beta \left(N_{1}\right)S_{a}E_{a}}{\partial S_{a}}\right]\right|}_{M_{a}^{*}}. \end{array}$$

Because

(40) $$\begin{array}{c} \frac{\partial \beta \left(N_{1}\right)S_{a}E_{a}}{\partial S_{a}}=\beta \left(N_{1}\right)E_{a}+\beta^{\prime }\left(N_{1}\right)S_{a}E_{a}\\ =\beta \left(N_{1}\right)E_{a}+\beta^{\prime }\left(N_{1}\right)\left(N_{1}-E_{a}\right)E_{a}\\ =\left[\beta \left(N_{1}\right)+\beta^{\prime }\left(N_{1}\right)N_{1}-\beta^{\prime }\left(N_{1}\right)E_{a}\right]E_{a}\\ =\left\{{\left[\beta \left(N_{1}\right)N_{1}\right]}^{\prime }-\beta^{\prime }\left(N_{1}\right)E_{a}\right\}E_{a}\\ \geq 0, \end{array}$$

and (∂*β*(*N*_1_)*S*_*a*_*E*_*a*_/∂*E*_*a*_)=*β*(*N*_1_)*S*_*a*_+*β*′(*N*_1_)*S*_*a*_*E*_*a*_, then [*ω*_1_ − ((∂*β*(*N*_1_)*S*_*a*_*E*_*a*_)/(∂*E*_*a*_))]|_*M*_*a*_^*∗*^_=((*aA*)/*E*_*a*_) − *β*′(*N*_1_)*S*_*a*_*E*_*a*_ > 0 for the reason that *β*′(*N*_1_) < 0. Therefore, $\text{det}\left(\left(\partial f/\partial x\right)\left(\bar{x}\right)\right)<0$. Condition (5) is satisfied.

Therefore, all the conditions of Lemma 4.1 are satisfied and *M*_*a*_^*∗*^ is globally asymptotically stable if *μ* ≥ ((∂*β*(*N*_1_)*S*_*a*_*E*_*a*_)/(∂*E*_*a*_)). It directly determines that *M*^*∗*^ is globally asymptotically stable if *μ* ≥ ((∂*β*(*N*_1_)*S*_*a*_*E*_*a*_)/(∂*E*_*a*_)) by the theory of limit system. The following conclusion is obtained.

**Theorem 6 .**

*M* ^*∗*^ is globally asymptotically stable if *μ* ≥ ((∂*β*(*N*_1_)*S*_*a*_*E*_*a*_)/(∂*E*_*a*_)).

It means that the endemic will arise if the self-healing rate or the cure rate of *E*_*a*_ is not less than the change rate of the incidence function *β*(*N*_1_)*S*_*a*_*E*_*a*_ with respect to *E*_*a*_. In other words, the endemic is very likely to occur. Therefore, in reality, we choose to kill all the poultry in an outbreak region for the sake of protecting the public health, suggesting that it is very important to seek strategies for disease prevention and control.

## 5. Simulations and Explanations

### 5.1. Investigations of Disease Control Measures

In this section, we will carry out simulations in purpose of investigating disease control measures.

Firstly, we will present the suitable functions *g*_1_(*E*_*a*_)*S*_*h*_, *g*_2_(*V*)*S*_*h*_, and *β*(*N*_1_)*S*_*a*_*E*_*a*_ in the need of simulations. In an ecological model, function ((*βSE*)/(1+*αE*)) is often used to express the saturated contact behaviour when the population is large and compact. Here, we choose *β*(*N*_1_)*S*_*a*_*E*_*a*_=((*βS*_*a*_*E*_*a*_)/(1+*αE*_*a*_)) due to the concentrated feeding mode in poultry industry. *β* embodies the transmission rate; *α* measures the inhibition rate. And we take the type of functions *g*_1_(*E*_*a*_)*S*_*h*_=(*η*_1_*E*_*a*_*S*_*h*_/1+*νE*_*a*_^2^) and *g*_2_(*V*)*S*_*h*_=((*η*_2_*VS*_*h*_)/(1+*νV*^2^)) that were introduced in [26], where *g*_*i*_(0)=0 and *g*_*i*_′(*x*)=((*η*_*i*_(1 − *νx*^2^))/(1+*νx*^2^)^2^), *i*=1,2, which satisfy the above corresponding modelling assumptions in Section 3. *η*_1_*E*_*a*_ and *η*_2_*V* measure the infection forces of the disease; *η*_*i*_, *i*=1,2 are the effective coefficients of the infection. 1+*νE*_*a*_^2^ and 1/(1+*νV*^2^) describe the psychological or the inhibitory effect for the behaviour changes of the susceptible people advocated by the authorities and the media when the epidemic deepens. *ν* expresses the inhibitory effect. Then, we will simulate the following system:

(41) $$\begin{array}{c} \left\{\begin{array}{c} \frac{dS_{a}}{dt}=\left(1-a\right)A-\frac{\beta S_{a}E_{a}}{1+\alpha E_{a}}-dS_{a}+\mu E_{a},\\ \frac{dE_{a}}{dt}=aA+\frac{\beta S_{a}E_{a}}{1+\alpha E_{a}}-dE_{a}-\xi E_{a}-\mu E_{a},\\ \frac{dV}{dt}=\varepsilon E_{a}-pV-qV,\\ \frac{dS_{h}}{dt}=B-\frac{\eta_{1}E_{a}S_{h}}{1+\nu E_{a}^{2}}-\frac{\eta_{2}VS_{h}}{1+\nu V^{2}}-\rho S_{h},\\ \frac{dI_{h}}{dt}=\frac{\eta_{1}E_{a}S_{h}}{1+\nu E_{a}^{2}}+\frac{\eta_{2}VS_{h}}{1+\nu V^{2}}-\delta I_{h}-\rho I_{h}-\gamma I_{h},\\ \frac{dR_{h}}{dt}=\gamma I_{h}-\rho R_{h}. \end{array}\right. \end{array}$$

Secondly, we need to estimate parameters. We measure time *t* in days. In Chinese poultry industry, commercial poultry is usually kept for 50 ~ 60 days. Hence, we get *d*=1/50=0.02 days^−1^. For an outbreak region, within 3 km of human cases, its population can refer to a small town, about 50000. There are 15 ~ 20 large farms in towns of this size, and a large farm often has 10000 chickens. Then, we set the total poultry population *N*_1_=150000 and the total human population *N*_2_=50000. Then, *A*=150000/50=3000 per day. And the known average lifespan is 75 years old in China in 2017. Therefore, *ρ*=1/(75 × 365)=3.65 × 10^−5^ days^−1^. We will take *B*=500, where 500 denotes the recruited population due to the mobile modern lifestyle, which is assumed as one percent of the local population.

Statistics for death cases from the WHO indicates that the mean duration of human infections is 6 ~ 7 days; therefore, we take the mean value *δ*=0.15 days^−1^. Referring to Tuncer and Martcheva [35], we assume that the virus-carried poultry shed virus into the environment at a rate *ε*=10^−4^, the virus in the environment decays at the rate *p*=*e*^−0.55^, the poultry-to-poultry transmission rate *β*=8 × 10^−5^ per individual per day, and the poultry-to-human transmission rate *η*_1_=0.2/(100 × *N*_1_) ≈ 1.3 × 10^−8^ per individual per day. We assume that the coefficient of transmission rate of the virus in the environment to human is *η*_2_=*η*_1_/10=1.3 × 10^−9^ per unit per day. Referring to Samsuzzoha et al. [36], we assume *γ*=0.16 per day. The incidence rate of *E*_*a*_ for case of HPAI is *ξ*=0.2 per day [37] and *ξ*=0 for case of LPAI. *α*, *μ*, *a*, *q*, and *ν* are the control parameters due to human interventions.

We assume that initially everyone is susceptible, thus *S*_*h*_(0)=50000 and *I*_*h*_(0)=0. And based on the report of China government that 20 percent of poultry carry the same virus as human infection cases in 2013 (China CDC), we assume *S*_*a*_(0)=0.80 × 150000=1.2 × 10^5^, *E*_*a*_(0)=0.20 × 150000=3 × 10^4^. The initial amount of virus in the environment measured in number of virions *V*(0)=10^−5^ [34].

In summary, the values of all fixed parameters are listed in Table 1.

**Figure 5:**
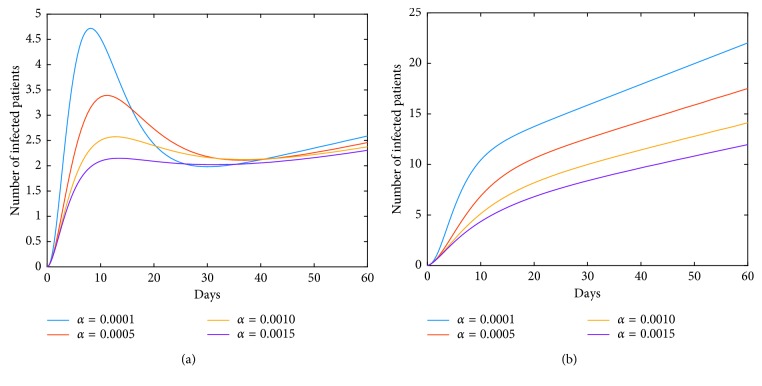
Impact of *α* on the human epidemic. *α* is the inhibition rate of transmission among poultry. The control effect is quite effective. Enhancing the inhibition rate in source poultry will contribute to the disease control in human society. A little effort will achieve good results. (a) Case of HPAI. (b) Case of LPAI.

**Figure 6:**
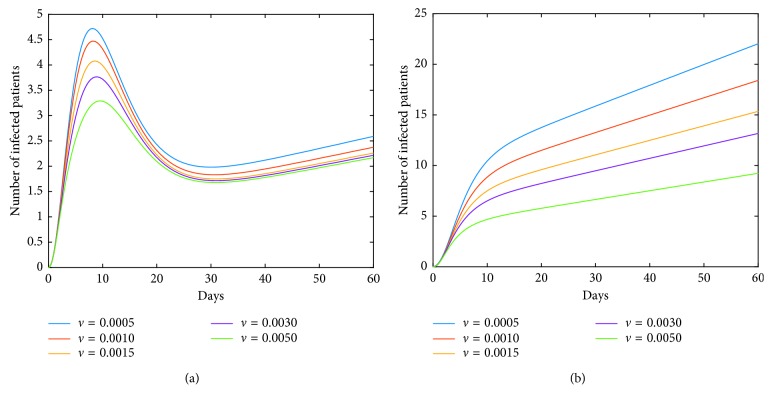
Impact of *ν* on the human epidemic. The inhibition rate *ν* mainly embodies the role of social psychological effects in human society. It is very unexpected that the control effect of *ν* alone is not ideal. (a) Case of HPAI. (b) Case of LPAI.

**Figure 7:**
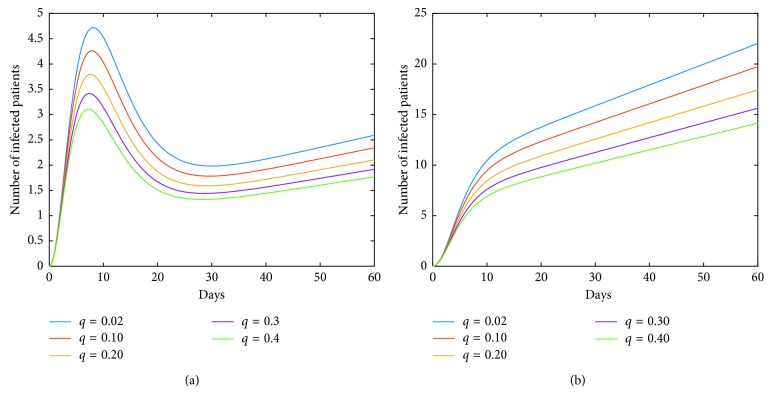
Impact of *q* on the human epidemic. *q* denotes the reduction rate of the virus in environment owing to people's disinfection measures. The control effect is positive. We should adhere to the cleanliness and disinfection of the relevant environment. (a) Case of HPAI. (b) Case of LPAI.

**Figure 8:**
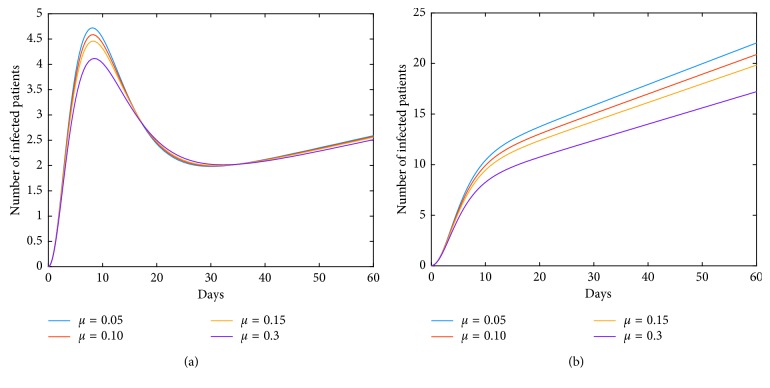
Impact of *μ* on the human epidemic. *μ* expresses the self-healing rate or the recovery rate of the virus-carried poultry with the help of people. Obviously, it has little success in the disease control. (a) Case of HPAI. (b) Case of LPAI.

**Figure 9:**
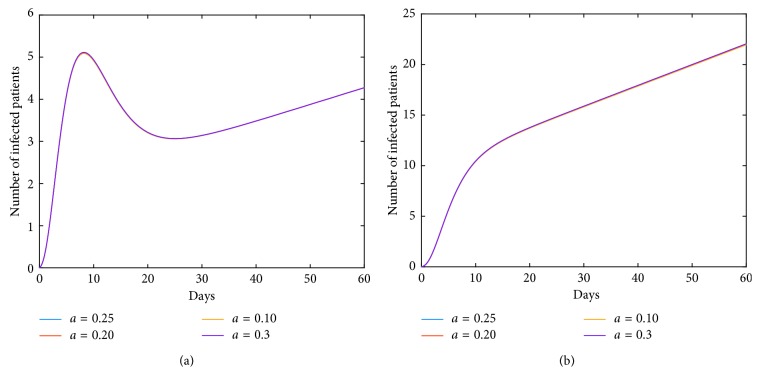
Impact of *a* on the human epidemic. *a* is the proportion of the virus-carried poultry in the recruitment. It can be seen that the varied *a* has no effect on the disease control in humans. (a) Case of HPAI. (b) Case of LPAI.

Now, we investigate control measures corresponding to parameters *α*, *μ*, *a*, *q*, and *ν*. It should be noted that other parameters are all fixed when studying the influence of one parameter. *α* expresses the inhibitory effect in poultry which can be achieved through people's efforts. We can see from Figure 5 that the impact of *α* for LPAI on humans will be more obvious than that for HPAI because the curve of *I*_*h*_(*t*) rises with time, and the number of infected patients also significantly increase. And it is quite effective of strengthening the inhibitory rate *α* for human infections with both HPAI and LPAI. The inhibitory effect of *ν* embodies the play of social psychological effects in human society which can be achieved through propaganda and education. It is obvious that the control effect of *ν* is not ideal, which is depicted in Figure 6. Combining up, it seems that it is more effective if we focus on the control of the source. *q* represents the reduction rate of the virus in environment due to people's disinfection measures. We can see from Figure 7 that the corresponding control effect is proportional to our efforts. *μ* denotes the possible self-healing rate which can be strengthened with the help of people, such as the actively preventive treatment. But Figure 8 shows that this effort has little success. *a* is the proportion of the infected poultry in the recruitment. Surprisingly, Figure 9 shows that controlling of the proportion *a* has no effect on human infections except the case of *a*=0. If *a*=0, based on theorems 4.2, 4.3, and 4.4, there is a disease-free equilibrium point *M*_0_, and it may be globally asymptotically stable, which means the disease is likely to be eradicated. That is, it does not make sense to do the input detection unless we can strictly eliminate the recruitment of virus-carried poultry.

Since the generally preventive treatment in poultry is not ideal for disease control, we will consider the specific vaccine strategy in poultry. We modify the parameter *μ* to the vaccination parameter *κ*. The modified model is as follows:

(42) $$\begin{array}{c} \left\{\begin{array}{c} \frac{dS_{a}}{dt}=\left(1-a\right)A-\frac{\beta S_{a}E_{a}}{1+\alpha E_{a}}-dS_{a}-\kappa S_{a},\\ \frac{dE_{a}}{dt}=aA+\frac{\beta S_{a}E_{a}}{1+\alpha E_{a}}-dE_{a}-\xi E_{a}-\kappa E_{a},\\ \frac{dV}{dt}=\varepsilon E_{a}-pV-qV,\\ \frac{dS_{h}}{dt}=B-\frac{\eta_{1}E_{a}S_{h}}{1+\nu E_{a}^{2}}-\frac{\eta_{2}VS_{h}}{1+\nu V^{2}}-\rho S_{h},\\ \frac{dI_{h}}{dt}=\frac{\eta_{1}E_{a}S_{h}}{1+\nu E_{a}^{2}}+\frac{\eta_{2}VS_{h}}{1+\nu V^{2}}-\delta I_{h}-\rho I_{h}-\gamma I_{h},\\ \frac{dR_{h}}{dt}=\gamma I_{h}-\rho R_{h}. \end{array}\right. \end{array}$$

**Figure 10:**
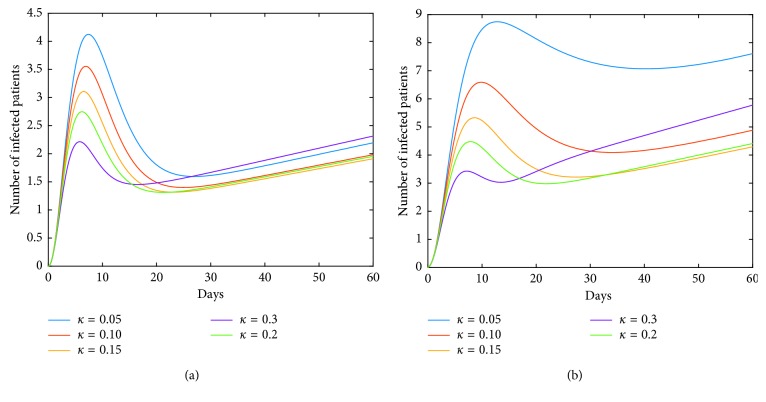
Impact of *κ* on the human epidemic. The vaccination rate *κ* can play important roles in disease control for both cases of HPAI and LPAI, especially in the case of LPAI. The important thing is that the vaccination measure in poultry changes the dynamics of human infections with LPAI virus, and the curve *I*_*h*_(*t*) is not monotonous. (a) Case of HPAI. (b) Case of LPAI.

**Figure 11:**
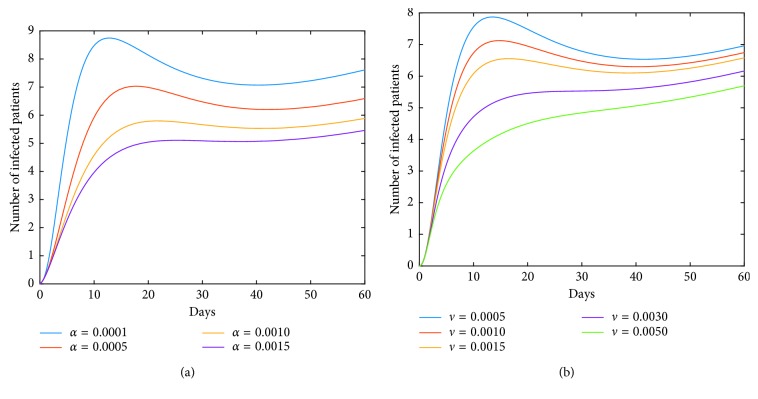
Roles of inhibition rates under the premise of implementing vaccine strategy in poultry for the case of LPAI. For the case of LPAI, under the premise of implementing vaccination in poultry, measures of inhibition rates all play well. Especially, the inhibition rate *ν* has played an effective role in the disease control, compared to the case in Figure 6. (a) Role of inhibition rate in poultry. (b) Role of inhibition rate in human society.

We can see from Figure 10 that the dynamics of human infections with LPAI is changed. It means that the vaccination measure in poultry is crucial for the control of human epidemic. That is, carrying out vaccination strategy in poultry enables the epidemic of human infections to fall back after a short-term peak, which contributes to the control of the epidemic. In addition, the vaccination measure for the case of HPAI is also quite effective. Furthermore, we can see from Figure 11 that controlling of both the two inhibition rates *α* and *ν* can be more effective under the premise of implementing vaccination in poultry for the case of LPAI. And the effect of social psychology, corresponding to parameter *ν*, shows its control effect while the effect is not ideal for the previous model (41).

**Figure 12:**
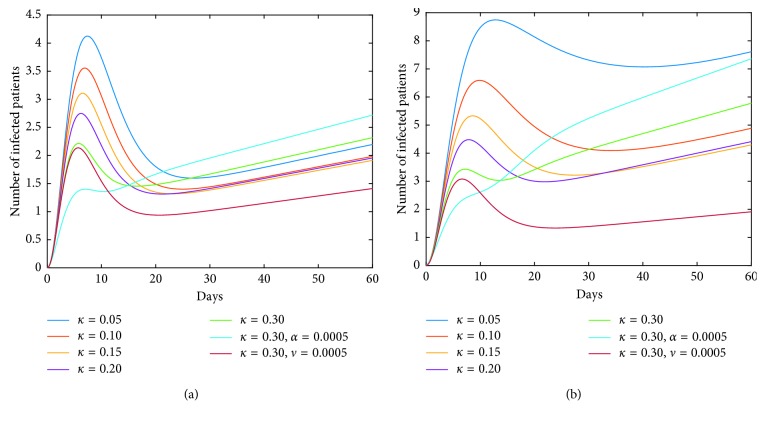
Collaboration of *κ* and *ν* (or *α*) on the human epidemic. The vaccine strategy in poultry will contribute to the disease control in humans. But for the larger *κ*, the epidemic is aggravated instead after a period of time. The inhibition rate in poultry can play a role in the short term and has a negative effect in the later stage, while the inhibition rate in human society plays an important role in disease control, especially in the later stage. Therefore, the simultaneous implementations of vaccine strategy in poultry and inhibition strategy in humans are the ideal control strategy. (a) Case of HPAI. (b) Case of LPAI.

On the other hand, we observe from Figure 10 that for some values of vaccination rate *κ*, such as *κ*=0.3, the number of infections rises to a greater value after a short period of time (about two weeks). For the reason that the greater value of *I*_*h*_(*t*) corresponds to the coordinate value of the equilibrium point *M*^*∗*^. In fact, the dynamic characteristic of the model determines that the positive equilibrium point *M*^*∗*^ is globally stable. That is, *I*_*h*_(*t*)⟶*I*_*h*_^*∗*^ as *t*⟶+*∞*. In other words, the influence of a parameter on the coordinate of the equilibrium point *M*^*∗*^ must also be taken into account when studying the corresponding role of this parameter. It means that it is not good to blindly strengthen the vaccination rate. Furthermore, we obtain from Figure 12 that simultaneous implementation of vaccine and inhibition strategies is the ideal control strategy. We can see that the inhibition rate *α* mainly plays its role in a week, and the inhibition rate *ν* plays its role after a week. Therefore, under the premise of vaccination in poultry, enhancing the inhibition rates *α* and *ν* will effectively control the epidemic.

### 5.2. Explanations of the Practical Measures

**Figure 13:**
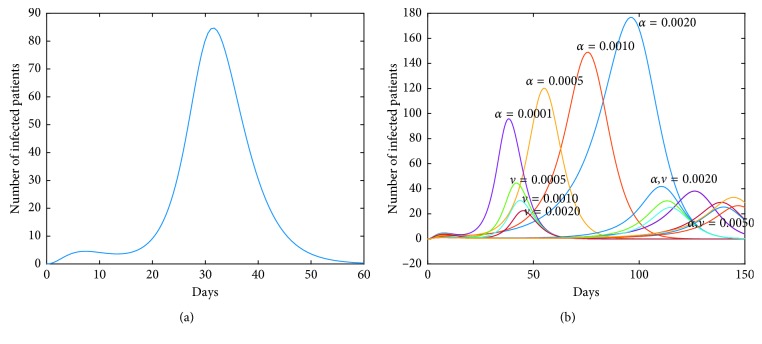
Measure of stopping live poultry transactions in the case of HPAI. *A*=0 corresponds to the stop of live poultry transactions. In this case, infections are suppressed in a short time but severe infections will occur soon after, and then the disease will be eliminated at last. The inhibition rate *α* will postpone the arrival of the peak; the inhibition rate *ν* will effectively decrease the peak value. By strengthening the two inhibition rates, the endemic in humans can be avoided. Such as, in the case of *α*, *ν*=0.0050, infections can be effectively suppressed within 4 months. (a) Dynamics of *A* = 0 (model (41)). (b) Impacts of *α* and *ν* in case of *A* = 0 on model (41) for HPAI.

**Figure 14:**
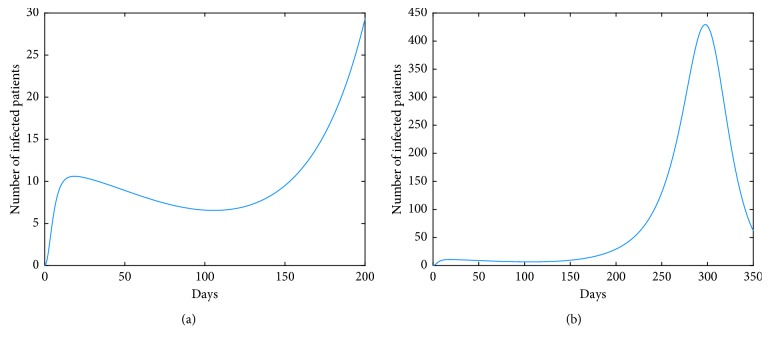
Dynamics of stopping live poultry transactions in the case of LPAI. Given *A*=0, the dynamics in the case of LPAI is similar to that of HPAI, but the detail description shows there is a four-month decline period after the first peak, and the second peak will appear in the tenth month after the outbreak.

**Figure 15:**
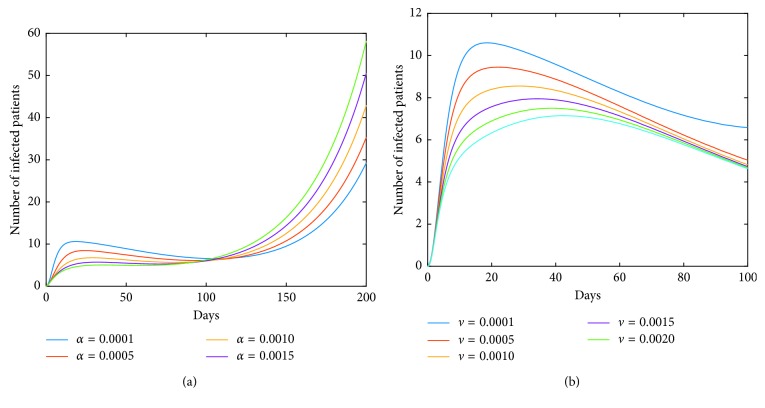
Impact of *α* and *ν* in the case of *A*=0 on model (9) for LPAI. In the case of human infections with LPAI, measure of stopping the live poultry together with strengthening inhibition rates *α* and *ν* will achieve the aim of controlling disease.

One of our countermeasures is to stop live poultry transactions. That is, *A*=0 in model (41). In this situation, we still maintain the normal production in farms, and poultry is kept for a longer time in farm. Simulation (Figure 13(a)) tells us that there is a short-term suppression but severe infections will occur three weeks later. But, from Figure 13(b), we find that strengthening the inhibition rate in humans can effectively reduce the peak value, but the increased inhibition rate in poultry intensifies rush-phase infections, and the arrival of the peak is postponed. It is worth noting that the delayed rush hour gives us time to control disease. Therefore, we can simultaneously enhance inhibition rates in both humans and poultry so that the peak value will be decreased and the peak time will be postponed, which is depicted in Figure 13(b). If we can postpone the peak time till six months later, we can avoid the virus's suitable temperature and climatic conditions; then, the endemic will not happen. At this point, for the case of human infections with LPAI, Figure 14 tells us that the severe infections will occur after 10 months. In fact, a wave of outbreak begins in October each year; it is summer after 10 months; therefore, the severe infections will not occur, and we will focus on the first peak, within 100 days. Furthermore, Figure 15 indicates that the inhibition rates *α* and *ν* all can effectively reduce the infections. Summing up, stopping live poultry transactions together with strengthening the two inhibition rates will achieve the aims of controlling disease and reducing economic losses simultaneously for human infections with both HPAI and LPAI.

Our another countermeasure is to kill all the poultry in the outbreak region. According to the above theoretical analysis, there is a disease-free equilibrium point *M*_0_ if *a*=0, and it is globally asymptotically stable if *Aβ*(*A*/*d*) ≤ *dω*_1_. But it is difficult to identify the virus-carried poultry because they do not show symptoms. That is, it is impossible to achieve *a*=0. And the disease-existence equilibrium point *M*^*∗*^ will be globally asymptotically stable if it exists. Therefore, in order to avoid the endemic we choose to cull all the poultry in the outbreak region. It means *A*=0, *S*_*a*_(0)=0, and *E*_*a*_(0)=0 in model (41); therefore, instantaneously *I*_*h*_(*t*)=0.

The third countermeasure is to disinfect the agricultural markets and farms. It will reduce the virus in environment. We have seen that the increasing *q* contributes to the disease control in Figure 7. What needs to be supplemented is that the places which need to be disinfected should also include parks and squares because there are many trees and birds tend to gather. Lastly, it is information disclosure and public education campaigns which will wake up public awareness and reduce the exposure, corresponding to the increased *ν* in model. Simulations show that it is a more effective measure under the premise of implementing vaccine strategy in poultry especially for human infections with the LPAI virus.

## 6. Conclusion

In early 2013 in China, the first case of human infection with the A H7N9 virus was confirmed, and then a new wave of outbreak occurred in autumn and winter every year. In this paper, we firstly investigate the temporal characteristics and the spatial distributions of these outbreaks. These results tell us that the most severe outbreak occurred in the fifth wave, followed by the second wave and the third wave. And the infection center moved from Yangtze River Delta area to the southeast coastal area, and subsequently the infected regions were expanded to most areas of mainland China.

Based on these distributions and other facts, a SEV-SIR avian-human eco-epidemiological model was established to characterize the two populations infected by an avian influenza virus. In this model, the constant recruitment, the general incidence functions, the factor of fully killed infected poultry, and the virus in environment are considered. The dynamic analysis was presented in Section 4. Firstly, the biological validity that the system is ultimately uniform bounded and dissipative was verified. If we can strictly eliminate the virus-carried poultry in the recruitment, there is a disease-free equilibrium point *M*_0_. And if we can also try to achieve *Aβ*(*A*/*d*) ≤ *dω*_1_, *M*_0_ is globally asymptotically stable. It means disease will be extinct at last. But it is not realistic for the reason that *a* will not be 0 because it is hard to identify the virus-carried poultry from the healthy individuals. Therefore, the disease-existence equilibrium point *M*^*∗*^ exists, and it is globally asymptotically stable in general. The endemic is inevitable. In view of reducing the risk of public health, disease control is very significant. According to the simulations, we have the following conclusions:

1. For model (41), strengthening the inhibition rate *α* is the most effective control measure, followed by the disinfection rate *q*. Adjustment of the proportion *a* is invalid for disease control. The general prevention and treatment in poultry, reflected by parameter *μ*, has little effect on human infections. Strengthening the inhibition rate *ν* alone is not ideal for disease control. These signify that it is important to control the spread in the source population.
2. If we modify the general treatment measure to the vaccination strategy, that is, for model (42), we find that the dynamic behaviour in the case of human infections with the LPAI virus is changed completely, and the curve of *I*_*h*_(*t*) is not monotonous. There is a regression process after the short-term peak, and then it rises monotonically. It will contribute to the disease control because we will try our best to control disease before rising again. For model (42), both for LPAI and HPAI, the effective control measures are measures corresponding to parameters *α*, *ν*, and *q* in order. *a* is still invalid. It is noted that the inhibition effect in human society should be combined with the vaccine strategy in poultry to play its role effectively.
3. As inhibition rates, *α* is more effective than *ν*. And the role of inhibition rate *α* is mainly reflected recently, while the role of inhibition rate *ν* is mainly reflected in the medium and long term. It also explains the importance of controlling in the infection source and the delay effect of psychosocial response. But the collaboration of the vaccine strategy in poultry and the inhibition effect in humans can play an excellent role on the disease control.
4. We have the way to control disease and reduce economic losses simultaneously. We need to stop live poultry transactions so that *A*=0. At the same time, measures have been taken to increase the inhibition rates *α* and *ν* in the two populations, respectively. The corresponding simulations are displayed in Figure 13(b) and Figure 15. On other hand, in the presence of infectious disease transmitted between two populations, common knowledge may be changed although it is still important to implement control measures in the source population. That is, in some cases, strengthening the inhibition rate in poultry will intensify the epidemic which is depicted in Figure 13(b), but the combined actions of the two inhibition rates can achieve excellent control results. It means that controlling in poultry and in humans is equally important for human infections with avian influenza virus.
5. The dynamic behaviour of case *κ*=0.30 in Figure 12 hints that it needs to combine numerical simulation and theoretical analysis to find the right control measure. Based on the theoretical analysis, the dynamics of the system is entirely determined by the source-subsystem. But, simulations tell us that vaccine strategy in poultry together with the inhibition effect in humans can help us achieve excellent control effects.

Summing up, measures of vaccination in poultry or stopping live poultry transactions, supplemented by enhanced inhibition rates in the two populations and the disinfection of the environment, can stop the spread of the virus and control the epidemic. Vaccine strategy in poultry is particularly important for human infections with LPAI virus.

Since the beginning of 2017, the situation of H7N9 prevention and control is obviously severe. After an extensive research, the poultry H7N9 vaccination has been implemented across the country since the autumn of 2017. As a result, the H7N9 influenza epidemic of poultry has been effectively controlled. And the effective control of poultry H7N9 influenza has blocked the transmission of pathogens from poultry to humans. According to the statistics, from October 2017 to March 2018, only 3 cases of human infections with the A H7N9 avian influenza virus have been confirmed in mainland China. Practical experience and the above investigations all confirm the importance of the vaccination strategy in poultry. It may be the reason why there was no epidemic of human infections with the A H7N9 in the autumn and winter of 2017.

## Figures and Tables

**Figure 16:**
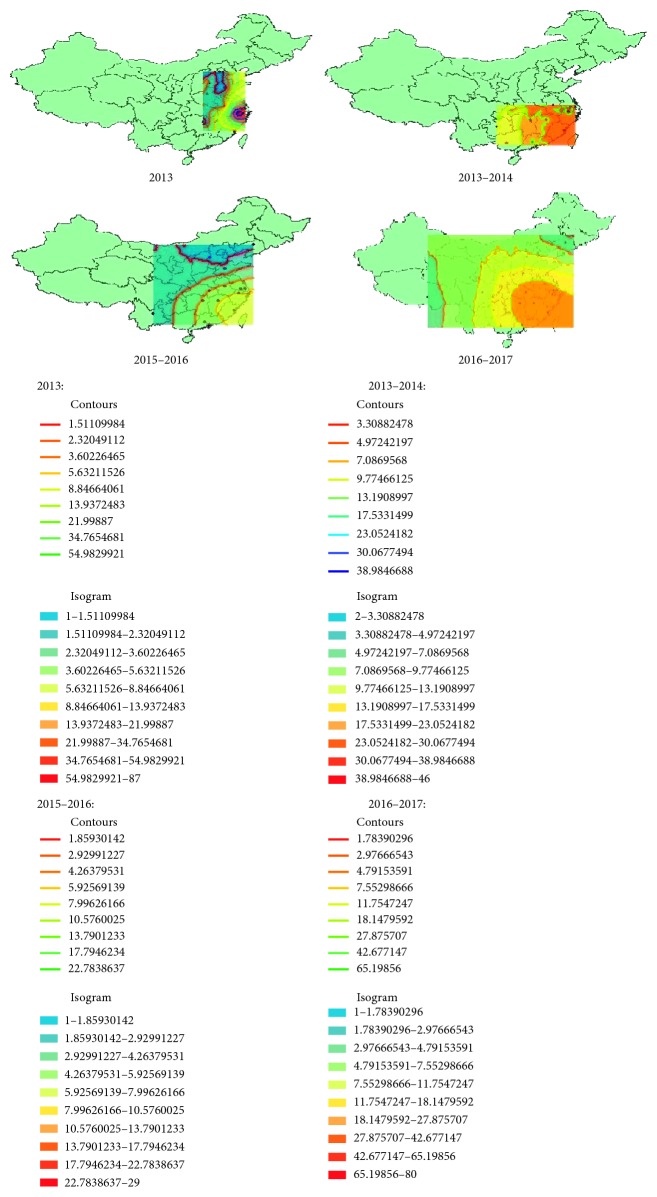


**Table 1:** Model parameters and values.

Parameter	Description	Value	Source
*A*	Constant recruitment rate of poultry	6 × 10^4^	Deduced
*B*	Constant recruitment rate of people	500	Assumed
*d*	Natural death rate of poultry	0.02	Deduced
*ρ*	Natural death rate of people	3.65 × 10^−5^	Deduced
*δ*	Additional death rate due to disease	0.15	Deduced
*ε*	Shedding rate of virus to environment	10^−5^	[35]
*p*	Decay rate of virus in environment	*e* ^−0.55^	[35]
*β*	Transmission rate of poultry-to-poultry	8 × 10^−5^	[35]
*η*_1_	Transmission rate of poultry-to-human	1.3 × 10^−8^	[35]
*η*_2_	Transmission rate of virus in environment to human	1.3 × 10^−9^	Assumed
*γ*	Recovery rate of human cases	0.16	[36]
*ξ*	Incidence rate of *E*_*a*_	0 (LPAI) or 0.2 (HPAI)	[37]

## Data Availability

The data of number of confirmed human cases are collected from the WHO-Disease Outbreak News (DONs). The spatial data of the locations of the cases used to depict the spatial distributions are obtained through Baidu map coordinates. The data of parameter values used to implement the sensitivity analysis have been included within the article.

## Appendix

Spatial distributions with data identifications are shown in Figure 16.
